# Identifying genetic lineages through shape: An example in a cosmopolitan marine turtle species using geometric morphometrics

**DOI:** 10.1371/journal.pone.0223587

**Published:** 2019-10-07

**Authors:** Rocío Álvarez-Varas, David Véliz, Gabriela M. Vélez-Rubio, Alejandro Fallabrino, Patricia Zárate, Maike Heidemeyer, Daniel A. Godoy, Hugo A. Benítez

**Affiliations:** 1 Departamento de Ciencias Ecológicas, Facultad de Ciencias, Universidad de Chile, Santiago, Chile; 2 Núcleo Milenio de Ecología y Manejo Sustentable de Islas Oceánicas (ESMOI), Departamento de Biología Marina, Universidad Católica del Norte, Coquimbo, Chile; 3 Karumbé NGO, Montevideo, Uruguay; 4 Centro Universitario Regional del Este (CURE), Sede Rocha, Universidad de la República, Rocha, Uruguay; 5 Departamento de Oceanografía y Medio Ambiente, Instituto de Fomento Pesquero, Valparaíso, Chile; 6 Centro de Investigación en Biología Celular y Molecular (CIBCM), Universidad de Costa Rica, San Pedro, San José, Costa Rica; 7 Centro de Investigación en Ciencias del Mar y Limnología (CIMAR), Universidad de Costa Rica, San Pedro, San José, Costa Rica; 8 Asociación para la Conservación Integral de Recursos Naturales Equipo Tora Carey (ETC), El Jobo, La Cruz, Guanacaste, Costa Rica; 9 Coastal-Marine Research Group, Institute of Natural and Mathematical Sciences, Massey University, Auckland, New Zealand; 10 Departamento de Biología, Facultad de Ciencias, Universidad de Tarapacá, Arica, Chile; Sao Paulo State University (UNESP/FCL/Assis), BRAZIL

## Abstract

The green turtle (*Chelonia mydas*) is a globally distributed marine species whose evolutionary history has been molded by geological events and oceanographic and climate changes. Divergence between Atlantic and Pacific clades has been associated with the uplift of the Panama Isthmus, and inside the Pacific region, a biogeographic barrier located west of Hawaii has restricted the gene flow between Central/Eastern and Western Pacific populations. We investigated the carapace shape of *C*. *mydas* from individuals of Atlantic, Eastern Pacific, and Western Pacific genetic lineages using geometric morphometrics to evaluate congruence between external morphology and species’ phylogeography. Furthermore, we assessed the variation of carapace shape according to foraging grounds. Three morphologically distinctive groups were observed which aligned with predictions based on the species’ lineages, suggesting a substantial genetic influence on carapace shape. Based on the relationship between this trait and genetic lineages, we propose the existence of at least three distinct morphotypes of *C*. *mydas*. Well-defined groups in some foraging grounds (Galapagos, Costa Rica and New Zealand) may suggest that ecological or environmental conditions in these sites could also be influencing carapace shape in *C*. *mydas*. Geometric morphometrics is a suitable tool to differentiate genetic lineages in this cosmopolitan marine species. Consequently, this study opens new possibilities to explore and test ecological and evolutionary hypotheses in species with wide morphological variation and broad geographic distribution range.

## Introduction

Marine turtles are migratory species with a complex life history. Their life cycle includes adult migrations from foraging grounds to generally distant breeding areas, and ontogenetic changes that affect the distribution of juveniles throughout a variety of marine habitats [1]. Natal homing behavior, wherein turtles return to their region of origin for mating and nesting, results in breeding stocks that are genetically differentiated. Foraging grounds harbor individuals from multiple natal origins [1, 2].

The green turtle (*Chelonia mydas*) has a circumglobal distribution including tropical, subtropical and temperate waters of the Pacific, Atlantic and Indian Oceans [3]. Like other globally distributed marine species, the evolutionary history of *C*. *mydas* has been molded by a sequence of isolation events generated by geological, oceanographic and climate changes [2, 4, 5]. The global phylogeography of the species indicates that the rise of the Panama Isthmus about 3.5 million years (Mya) separated Atlantic from Pacific green turtle populations [2]. In the Pacific Ocean, Dutton et al. (2014) [6] suggested a complex population genetic structure in *C*. *mydas*, with a distinct phylogeographic break between Western and Central/Eastern Pacific populations. The split between the Central/Eastern Pacific and Western Pacific lineages was estimated at around 0.34 Mya, suggesting that the Eastern Pacific was recently colonized [6]. Although Dutton et al. (2014) [6] demonstrated that populations of the Central and Eastern Pacific are reproductively isolated from those of the Western Pacific region, evidence from foraging areas in these regions suggests that both juveniles and adults disperse occasionally throughout the Pacific to feed [7–10]. Namely, individuals with Western Pacific natal origin (Western Pacific genetic lineage) have been observed feeding in Eastern Pacific sites and vice versa.

Morphological variability (e.g. carapace length, carapace scute pattern, flipper size, skull morphology) have been described for different *C*. *mydas* populations at a global scale [3, 11, 12, 13]. However, only two morphotypes for the species have been widely recognized: a light morphotype with nesting colonies globally distributed (Atlantic, Indian and Western Pacific) and a black morphotype with its natal origin restricted to the Eastern Pacific [8, 14, 15]. The light-colored morphotype is characterized by having an oval carapace varying from cream-yellow to earth colors [7, 8, 16]. The black form has a conical shaped carapace, almost black, plain or spotted [7, 15–17]. Despite the observation that black turtles are different from their counterparts anywhere in the world, the extensive morphological variation of *C*. *mydas* specially associated with its coloration has made the identification of both morphotypes difficult in foraging areas of the Pacific Ocean where they exist sympatrically [18–20].

Genetic studies using control region of mtDNA have corroborated transoceanic migratory events of *C*. *mydas* across the Pacific Ocean, and have reported congruence between haplotypes and morphotypes (i.e. light morph/Western Pacific lineages and black morph/Eastern Pacific lineages) [7, 9, 10]. However, a recent study found inconsistencies between control region haplotypes and morphotypes, where coloration was variable but carapace shape was consistent with morphotypes visual descriptions [20]. In this context, tools such as geometric morphometrics (GM), which quantifies variation in the shape of a structure [21], may be useful to classify individuals beyond their coloration. GM quantifies variation in the shape of objects, after the effects of nonshape variation (position, orientation and scale) have been mathematically held constant [21, 22]. Previous studies using this tool have successfully differentiated intra-specific lineages and morphotypes or ecotypes in several taxa [23, 24]. For marine turtles, the few published studies to date have focused on sexing hatchlings [25, 26], the relationship between incubation duration and carapace shape variation [27], variation in skull morphology [28] and variation of allometric and non-allometric shape [29].

Previous evidence indicates the carapace shape in chelonians possesses a significant heritable genetic component [30, 31, 32], and some studies have reported an association between phylogeographic differentiation and shell shape variation both tortoises and freshwater turtles [32, 33, 34].

Given the evolutionary history of *C*. *mydas* in the Atlantic and Pacific basins, and the heritability of this trait, in our study we expect to find congruence between carapace shape variation and genetic lineages of this species. We hypothesized the presence of three morphological distinctive groups: Atlantic Group, Eastern Pacific Group and Western Pacific Group. Here, we examined the morphological variation of *C*. *mydas* according to genetic lineages or natal origins (Atlantic, Eastern Pacific and Western Pacific) and also according to foraging grounds (Uruguay, Costa Rica, Galapagos-Ecuador, Chile and New Zealand).

## Materials and methods

### Ethic statements

All procedures involving animals were carried out in accordance with approved guidelines and protocols under permits issued by national agencies from all countries involved in this study. Details on permits are described below.

### Study area and data collection

Our study included *C*. *mydas* foraging grounds located in the South Western Atlantic (Uruguay), Eastern Pacific (from north to south: Costa Rica, Galapagos-Ecuador and Chile) and South Western Pacific regions (New Zealand). Specific locations are described below:

#### Uruguay

The Coastal-Marine Protected Area of Cerro Verde (CMPA; 33.93° S, 53.50° W) is located in north-eastern Uruguay. Juvenile turtles (n = 197) were captured alive using nets during the warmer months (December to April) between 2012 and 2016. The capture method is described in [35]. Captures were conducted by Karumbé NGO technicians and were authorized by the Fauna Department-Ministry of Cattle, Agriculture and Fishing of Uruguay (license no. 073/08, 323/11, 12/14), and the Fauna Division-Ministry of Housing, Territorial Planning and Environment of Uruguay (DF 141/16).

#### Costa rica

Matapalito Bay (10.93°N, 85.78°W) is a small inlet of the Santa Elena Peninsula located in north-western Costa Rica. Juveniles and adult turtles (juveniles, n = 22; adults, n = 20) were captured using nets between 2012 and 2017 as described in [36]. Field work and sampling were authorized by the Guanacaste Conservation Area (ACG) of the Ministry of Environment, Energy and Telecommunications (MINAE).

#### Galapagos

The Galapagos Archipelago (~00.66°S, 90.55°W) is a group of islands of volcanic origin located about 1,000 km west of mainland Ecuador. Juvenile and adult turtles n = 74; and n = 5, respectively) were captured with nets and by hand in the Galapagos foraging grounds between 2004 and 2005. Capture methods are described in [8]. Research permits were provided by the Galapagos National Park.

#### Chile

Bahía Salado (27.68°S, 70.98°W) is a bay located in the Atacama Region in northern Chile (mainland Chile). Juvenile turtles (n = 9) were captured using nets during spring 2014 and summer 2018. The capture method is described in [37]. Captures were authorized by the Chilean Sub-Secretariat of Fishing (SUBPESCA, by its Spanish abbreviation), through a Research Capture Permit granted in April 2013 (Exempt Resolution no. 917) and renewed in July 2014.

#### New Zealand

The study area encompassed the inshore waters of North Island, extending from Cape Reinga (34.41°S, 174.66°E) south to Opotiki, in the Bay of Plenty (37.98°S, 177.28°E). Live and dead stranded juvenile turtles (n = 25) were collected for examination between 2012 and 2017. All individuals included in this study exhibited good body condition and the cause of stranding or death was not associated with an underlying pathology (most died by interaction with fishing gear). In addition, all dead individuals were fresh (with presence of eyes, all head scales and with carapace scutes intact) [38]. Collection specifications are described in [9]. Collections were authorized by the Department of Conservation of New Zealand (authorization no. AK 30931-FAU and 52128-FAU).

### Genetic lineage determination

In order to differentiate genetic lineages in Pacific *C*. *mydas* foraging grounds, mtDNA control region haplotypes were identified for each individual using primers LCM15382 (5'- GCTTAACCCTAAAGCATTGGO3') and H950g (5'GTCTCGGATTTAGGGGTTTGO3') designed by [39]. These data were obtained according to the project’s specific frameworks undertaken in each country in collaboration with external researchers (Costa Rica, Heidemeyer et al. unpublished data; Galapagos, Dutton & Zarate unpublished data, Chile, [37]; New Zealand, [40]). Thus, for the purposes of this paper, only the lineage (natal origin) corresponding to the Eastern or Western Pacific was indicated (not the specific haplotype; S1 Table). Two divergent evolutionary lineages for *C*. *mydas* have been described in the Atlantic/Mediterranean region, the “northern lineage” and the “southern lineage” [41–43]. The southern lineage encompasses the eastern Caribbean, South Atlantic and West African rookeries [41]. Given that about 90% of green turtles foraging in Uruguayan waters have their natal origin in the South Atlantic region [44], turtles from Uruguay of this study were classified into “Atlantic southern lineage”.

### Shape analysis

**Fig 1:**
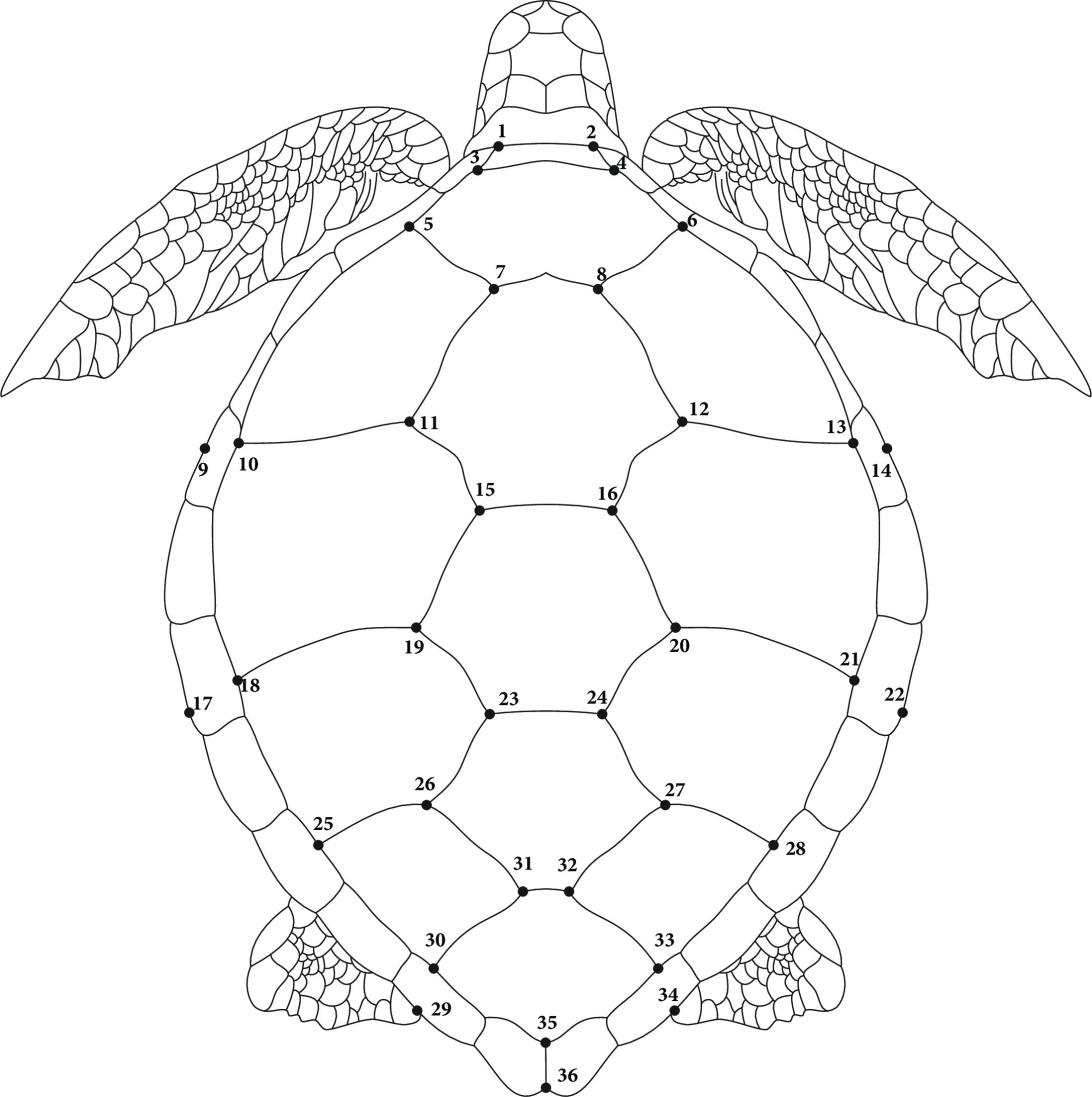
Representation of the 36 landmarks identified on *Chelonia mydas* carapace.

Geometric morphometric analyses included 352 *C*. *mydas* individuals from five foraging grounds: 197 with Atlantic natal origin (Atlantic genetic lineage-AGL, Uruguay); 105 with Eastern Pacific natal origin (Easter Pacific genetic lineage-EPGL, Costa Rica, Galapagos, Chile and New Zealand) and 50 with Western Pacific natal origin (Western Pacific genetic lineage-WPGL, Costa Rica, Galapagos and New Zealand) (S1 Table). GM analysis focused on variation in carapace shape, and was performed using dorsal photographs of individuals of distinctive genetic lineages and foraging grounds. All photographs were obtained using a reference scale. Thirty-six landmarks (Fig 1) were digitized with TPS Dig 2.30 software [45]. Landmarks were obtained between specific carapace scutes and at the borders of the marginal scutes, projected from the closest lateral scute [29]. A Procrustes superimposition was applied to the landmark data in order to remove any non-shape elements.

A multivariate regression was carried out to determine the influence of size on shape (allometry) in the dataset using centroid size (size variable) as an independent variable and shape (Procrustes coordinates) as a dependent variable [46]. Furthermore, a permutation test using 10,000 iterations was performed to assess the significance of the influence of the size on shape.

Principal component analyses (PCA) were performed using the covariance matrices of shape variation and the average shape variation in genetic lineages and foraging grounds. To visualize shape average changes and their distribution in the averaged shape space a PCA scatterplot was performed. Additionally, in order to visualize the variation in carapace shape, the average carapace shape was rendered for each genetic lineage and foraging ground.

A canonical variate analysis (CVA) was performed to have a better graphical representation of the data and to discriminate groups based on carapace shape variation in different genetic lineages and foraging grounds. The CVA is a multivariate statistical method used to find the shape characters that best distinguish among groups of specimens. The results were reported as Procrustes distances and the respective p-values for these distances, after permutation tests (10,000 iterations).

A Procrustes ANOVA was carried out to assess the significance of the differences in carapace shape between genetic lineages and between foraging grounds. All analyses were performed using MorphoJ software [47]. For these analyses, data were pooled according to genetic lineage (Atlantic, n = 197; Eastern Pacific, n = 105 and Western Pacific, n = 50), and foraging ground (Uruguay, n = 197; Costa Rica, n = 42; Galapagos, n = 79; Chile, n = 9 and New Zealand, n = 25) (S1 Table).

## Results

### Carapace shape variation according to *C*. *mydas* genetic lineages

**Fig 2:**
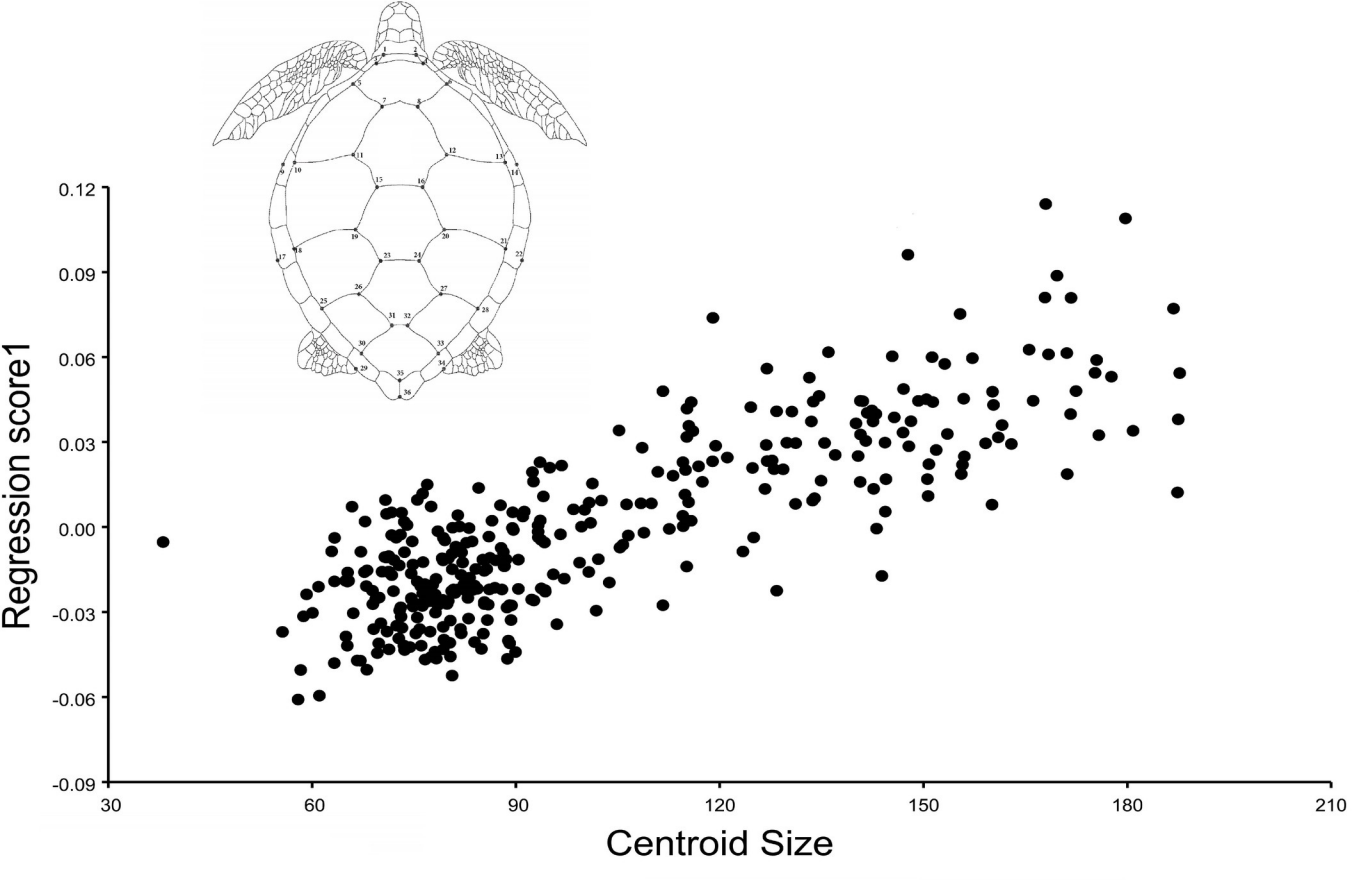
Multivariate regression of the carapace shape on carapace centroid size.

Multivariate regression showed a 21.6% of allometry with a significant permutation value (p-value = <0.0001) (Fig 2). Thus, a correction for allometry was performed and all the shape analyses (PCA and CVA) were carried out using the covariance matrix of the data corrected by size (data used from the residual of the multivariate regression). Given this allometric correction, a distinction between juveniles and adults was not performed in this study.

**Fig 3:**
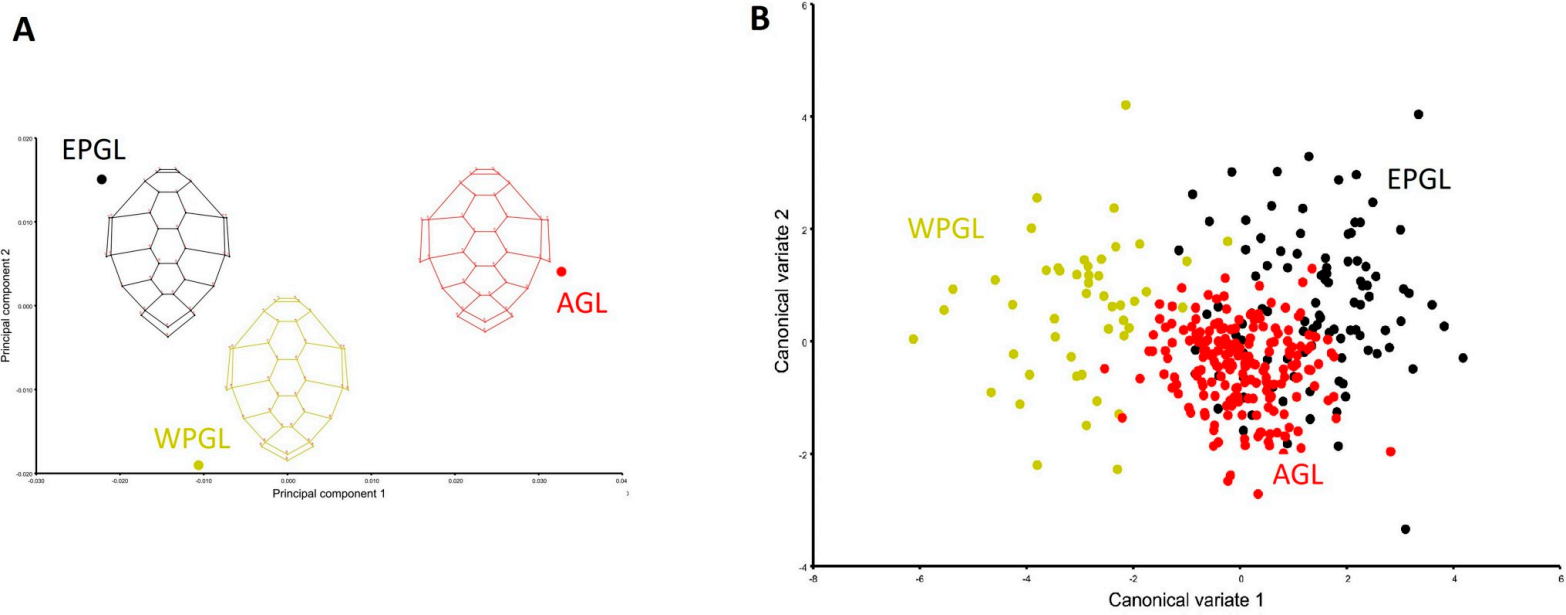
Difference in carapace shape between *Chelonia mydas* from different genetic lineages. (A) Principal component analysis of the average carapace shape (B) Scatterplot of first two axes of the canonical variate analysis. Eastern Pacific (EPGL): black; Western Pacific (WPGN): yellow, and Atlantic (AGL): red. * All the analyses have size effect removed.

The first three principal components (PC) accounted for 69.7% of shape variation (PC1 = 43.6%; PC2 = 13.4% and PC3 = 12.64%). The scatterplot of the PCA (S1 Fig) showed a central cloud of points from the AGL, in contrast to a higher dispersion of points between the two Pacific lineages (EPGL and WPGL). Average carapace shape based on genetic lineage clearly varied between groups (Fig 3A). The AGL exhibited a wider carapace, while, in contrast, an oval and triangular carapace shape were observed in the WPGL and EPGL, respectively. Differences in the second lateral scute were identified (landmarks 10–13, 15, 16 and 18–21), with an antero-posterior narrowing in the EPGL in comparison to the other two lineages. Moreover, the last vertebral scute (defined by landmarks 30–33 and 35) in turtles from the EPGL was longer, and all marginal scutes (landmarks 9–10, 13–14, 17–18, 21–22, 29–30, 33–36) were wider in turtles from the AGL.

The CVA was able to segregate all lineages in its first 2 axes: CV1 separated the WPGL, and CV2 the AGL and the EPGL (Fig 3B). The Procrustes ANOVA (Table 1) showed significant differences between genetic lineages which were confirmed after run paired permutation test between the Procrustes distances between groups (p-value = <0.0001; Table 2).

**Table 1:** Procrustes ANOVA performed to assess significance between genetic lineages and foraging grounds on both centroid size and shape of *Chelonia mydas*. Sums of squares (SS) and mean squares (MS) are in units of Procrustes distances (dimensionless).

**Centroid Size**					
**Effect**	**SS**	**MS**	**df**	**F**	**P (param.)**
Individual	89203.88671	265.48776	336	1514	0.0205
Genetic lineages	3708.08282	1854.0414	2	6.98	0.0011
Foraging grounds	11262.84389	2815.711	4	10.61	<0.0001
Residual	0.175368	0.175368	1	-	-
**Shape**					
**Effect**	**SS**	**MS**	**df**	**F**	**P (param.)**
Individual	0.77483232	6.783E-05	11424	3.48	<0.0001
Genetic lineages	0.0369254	0.000543	68	8.01	<0.0001
Foraging grounds	0.03741178	0.0002751	136	4.06	<0.0001
Residual	0.00550339	8.093E-05	68	-	-

Sums of squares (SS) and mean squares (MS) are in units of Procrustes distances (dimensionless).

**Table 2:** Results of the CVA analysis with Procrustes distances and their respective p-values between genetic lineages. Eastern Pacific (EPGL); Western Pacific (WPGN) and Atlantic (AGL).

	AGL	EPGL
**EPGL**	0.0546	-
	<0.0001	-
**WPGL**	0.0391	0.0362
** **	<0.0001	<0.0001

### Carapace shape variation according to foraging grounds in the Atlantic and Pacific Oceans

**Fig 4:**
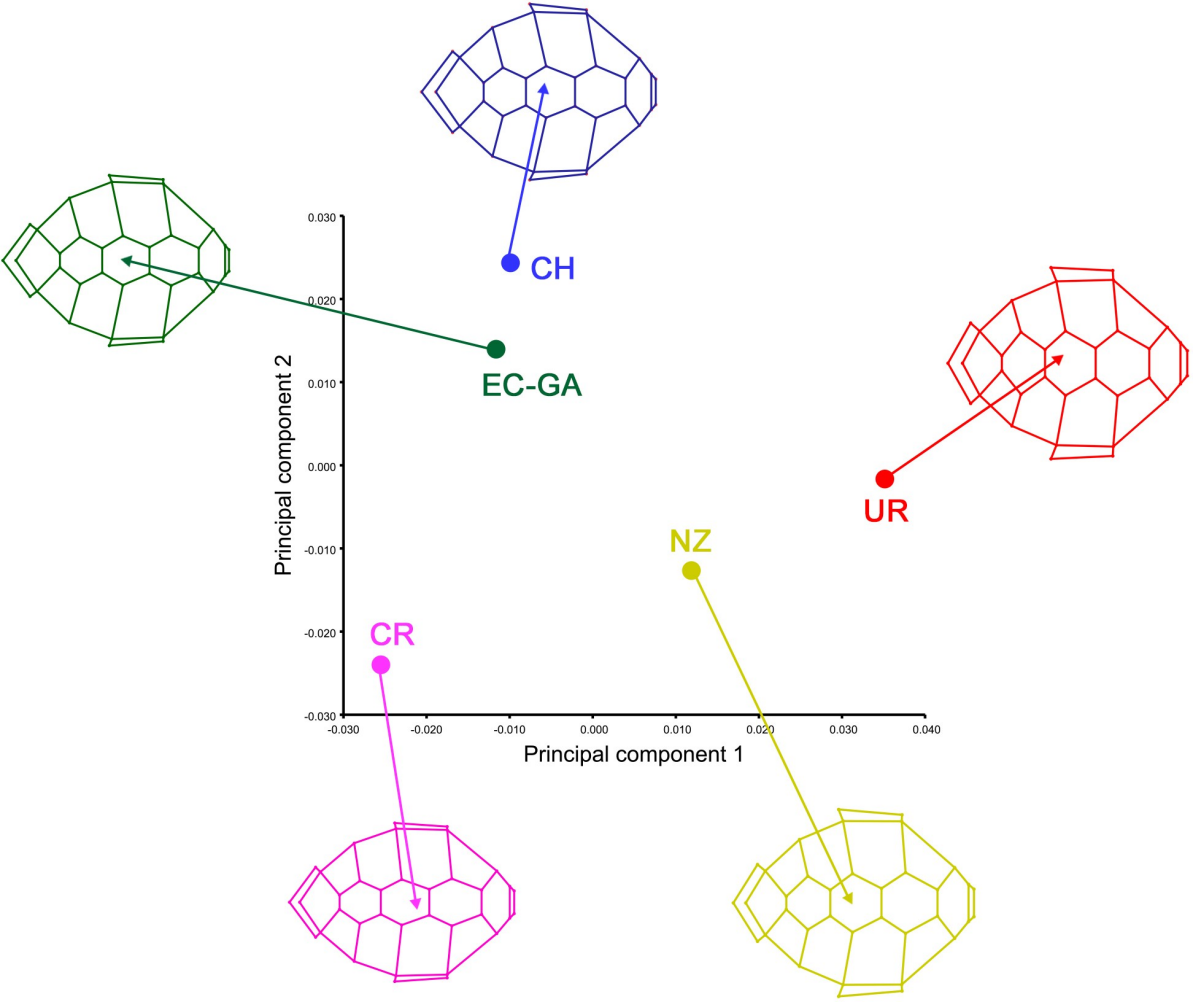
Principal component analysis of the average carapace shape of *Chelonia mydas* from foraging grounds. Uruguay: red; Costa Rica: pink; Galapagos, Ecuador: green; Chile: blue, and New Zealand: yellow.

**Fig 5:**
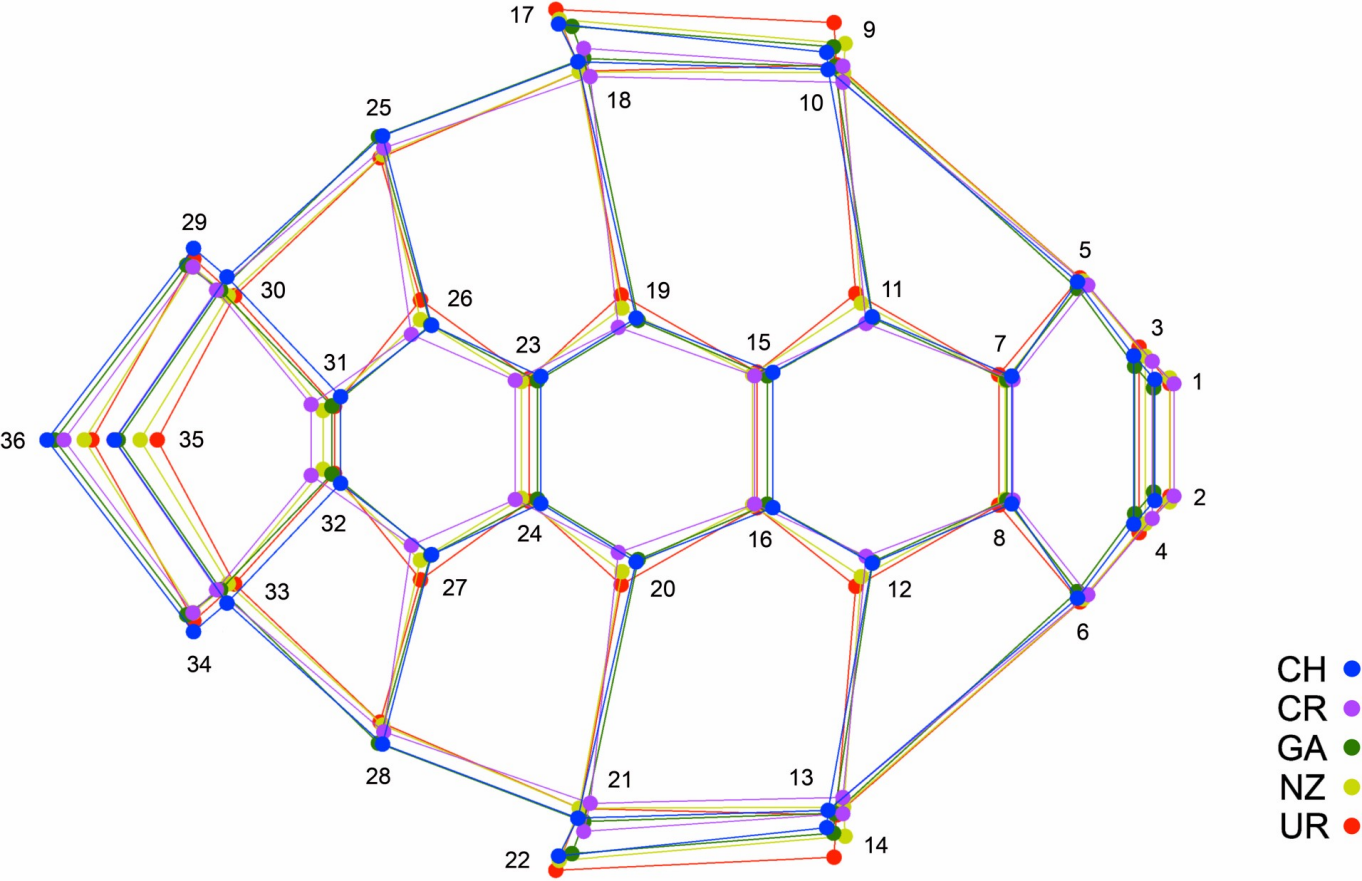
Wireframe representation of the carapace shape variation and their corresponding landmarks from foraging grounds. Uruguay: red; Costa Rica: pink; Galapagos, Ecuador: green; Chile: blue, and New Zealand: yellow.

Average carapace shape based on foraging ground varied between groups, being more similar between Chile and Galapagos and between New Zealand and Uruguay (Fig 4). New Zealand and Uruguay showed wider vertebral and marginal scutes and the last vertebral scute (defined by landmarks 30–33 and 35) shorter in comparison to turtles from Chile, Galapagos and Costa Rica (Fig 5).

**Fig 6:**
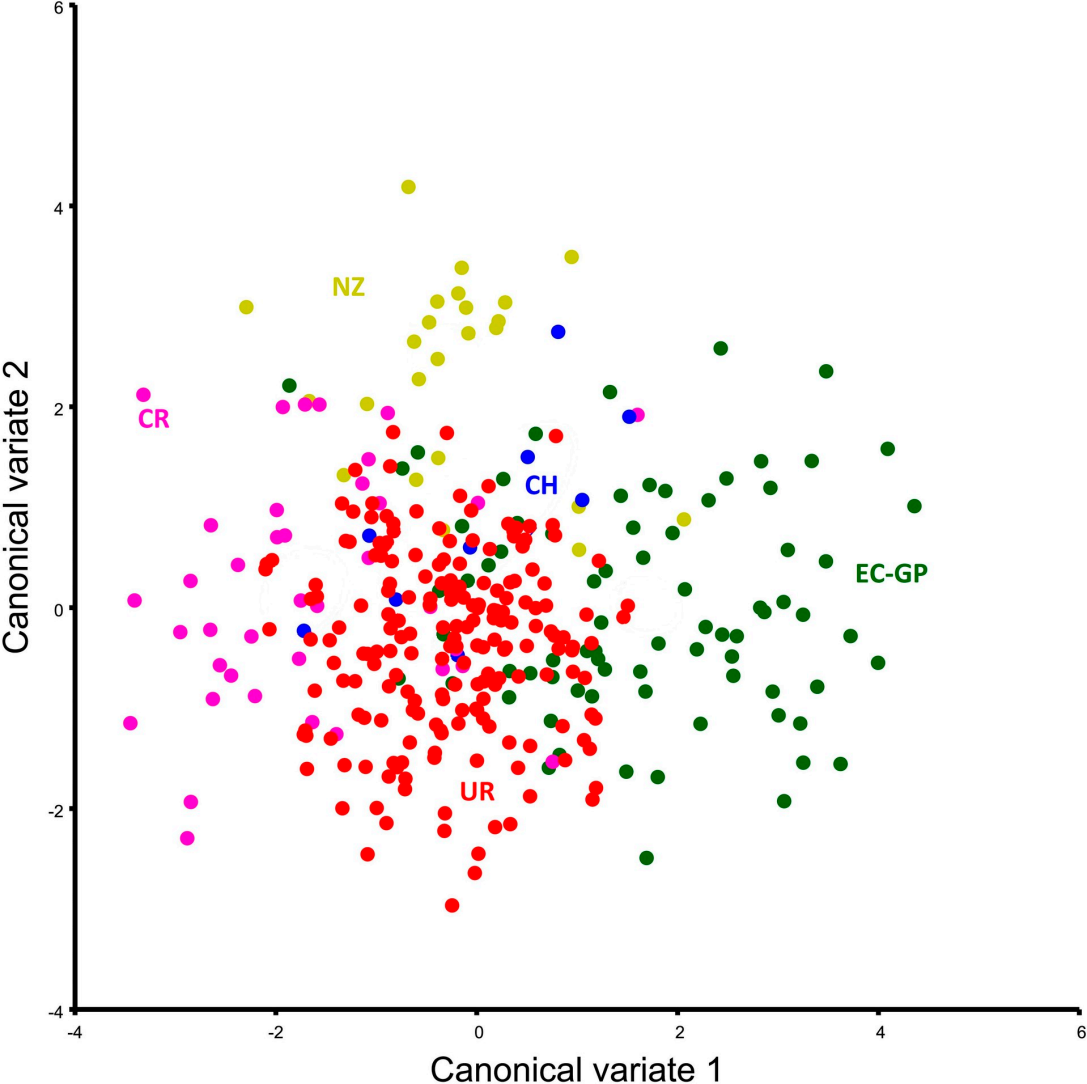
Difference in carapace shape between *Chelonia mydas* from different foraging grounds. **Scatterplot of first two axes of the canonical variate analysis.** Uruguay: red; Costa Rica: pink; Galapagos, Ecuador: green; Chile: blue, and New Zealand: yellow. *All the analyses have size effect removed.

The CVA separately grouped individuals from Uruguay, Costa Rica, Galapagos and New Zealand (Fig 6). In contrast, there was no clear differentiation of the Chilean group. Procrustes ANOVA showed significant differences between foraging grounds for both centroid size and shape (Table 1). Procrustes distances were significant between foraging grounds, except between Galapagos and Chile (p-value = 0.2811; Table 3).

**Table 3:** Results of the CVA analysis with Procrustes distances (below diagonal) and their respective p-values (above diagonal) between foraging grounds. Uruguay: UR; Costa Rica: CR; Galapagos, Ecuador: GA; Chile: CH and New Zealand: NZ.

	UR	CR	GA	CH	NZ
**UR**	-	<0.0001	<0.0001	<0.0001	<0.0001
**CR**	0.0647	-	<0.0001	0.0100	<0.0001
**GA**	0.0511	0.0430	-	0.2811	0.0002
**CH**	0.0530	0.0513	0.0232	-	0.0070
**NZ**	0.0288	0.0407	0.0381	0.0446	-

## Discussion

### Congruence between shape variation and evolutionary history of *C*. *mydas*

Our study showed differences of the carapace shape between turtles from the Atlantic and Pacific Ocean basins, and also among individuals within the Pacific Ocean. These variations at the geographical scale may be driven by historically changing geologic and climatic conditions. The Panama Isthmus closed off the Pacific-Atlantic connection about 3.5 Mya [2], and since then has played an important role in the divergence of Pacific and Atlantic clades of different marine turtle species including *C*. *mydas* [4, 48, 49]. In the Pacific Ocean, the East Pacific Barrier (EPB), a 5000 to 8000 km deep-water extension located east of Hawaii, has been described as one of the most important barriers for dispersal separating Eastern Pacific biota from the Central Pacific and Indo West-Pacific regions [50, 51]. Nevertheless, a recent study of *C*. *mydas* populations suggested that the Pacific region west of Hawaii has been a more significant barrier to gene flow than the EPB, and that the split between Central/Eastern and Western lineages in this species occurred about 340,000 years ago [6]. The uplift of the Panama Isthmus precedes the divergence in *C*. *mydas* described by Dutton et al. (2014) [6] in the Pacific Ocean (Eastern and Western Pacific lineages), and this is congruent with the degree of morphological differentiation observed in this study, where differences were more evident between basins (Atlantic-Pacific) than within the Pacific basin.

Natal homing behaviour has been demonstrated in most marine turtle species using mtDNA sequencing [2]. These maternally inherited markers show strong population structure among nesting colonies while nuclear loci reveal a contrasting pattern of male-mediated gene flow [2]. Particularly in *C*. *mydas*, studies confirm this reproductive behaviour for females and males; however, the geographic specificity of homing is uncertain, and it may vary for hundreds of kilometres among different populations [5, 6, 52]. In the Pacific Ocean, our results showed two distinctive morphological groups that were consistent with genetic lineages (EPGL and WPGL). This aligned with the natal homing theory that states turtles returning to their region of origin for mating and nesting, which influences the population’s genetic structure.

In summary, our study showed a parallel between carapace shape variation and the evolutionary history of *C*. *mydas*, as initially predicted, associated with a geographic barrier limiting gene flow between ocean basins (Panama Isthmus). In addition, it is possible this association may also be influenced by life history traits (i.e. natal homing) and oceanographic conditions (e.g. barrier west of Hawaii) that differentiate populations within the Pacific Ocean. Likewise, the congruence between phylogeography and morphology here, suggests a significant genetic influence on the carapace shape in *C*. *mydas* as reported in other chelonians [30, 31, 32].

### Association between grouping based on carapace shape and morphotypes of *C*. *mydas*

The East Pacific form of *C*. *mydas* tends to be distinguished by conical carapace shape and dark coloration [7, 15, 17]. Although genetic data do not support the evolutionary distinctiveness of the black morphotype, a population level-differentiation exists between this form and the lighter form (light morphotype) [4, 53]. Kamezaki & Matsui (1995) [13] examined geographic variation of skull morphology in *C*. *mydas* and they observed an exclusive distinction of black turtles (Galapagos nesting population), suggesting that due to its isolation, this group contains unique morphological characteristics. Later, Okamoto & Kamezaki (2014) [54] studied *C*. *mydas* foraging grounds in Japan (Pacific Ocean) and reported differences in carapace shape between black and light morphs, with a narrowing at the level of the eleventh marginal scute in black turtles, which remained consistent throughout growth. In this work, using geometric morphometrics, we evaluate the carapace shape of *C*. *mydas* and we observed that turtles with a Western Pacific origin (WPGL, putative “light morph”), exhibited an oval or more elongated carapace, while turtles with an Eastern Pacific origin (EPGL, “black morph”) exhibited a triangular (conical) carapace. These results are consistent with the visual descriptions of both morphotypes [7, 15–17]. Moreover, turtles from the EPGL exhibited a narrowing of the second lateral scute and an elongation of the last central scute (Fig 3A). On the other hand, grouping based on genetic lineages was found here, differentiating the Atlantic group, and two groups of the Pacific (Fig 3B). Such grouping did not correspond with the worldwide recognized assignation of morphotypes [7, 15, 17], due to our data showed the presence of a distinctive Atlantic morphotype that differs from the light morphotype that occurs in the Pacific Ocean (Fig 3).

Until now, differences in carapace shape between *C*. *mydas* from the Atlantic and Pacific lineages and intra-Pacific lineages had not been tested. Colour alone should not be considered as a diagnostic tool to distinguish between populations because this character is highly variable throughout the range of *C*. *mydas* [9, 55]. In fact, for this reason we did not include this character in our analysis.

Our results show that carapace shape could enable us to differentiate intraspecific genetic lineages in this cosmopolitan species. Based on these results, we propose the existence of at least three distinct morphotypes: Atlantic, Eastern Pacific and Western Pacific. Nevertheless, further research incorporating other evolutionary lineages (e.g. “northern lineage” from the Atlantic/Mediterranean) may provide more insight into carapace shape variation and the designation of other morphotypes, globally.

### Carapace shape variation through foraging grounds in the Pacific Ocean: Conservation implications

Green turtles have a circumglobal distribution with hundreds of nesting beaches and foraging grounds making up a complex network of migratory routes [1]. As described previously, a marked genetic differentiation has been observed between green turtle populations at a larger scale in the Pacific Ocean (corresponding to EPGL and WPGL [6]), which is most likely associated to oceanographic conditions and natal homing behavior. At a finer scale, using mitochondrial markers, genetic structuring has also been observed, which has been used to identify distinctive management units (MUs) [6, 10]. Management units or stocks correspond to populations that exchange so few migrants that are genetically distinct and demographically independent [56]. Specifically, in the Central and Eastern Pacific region, five MUs have been designated: Northwest Hawaii, Revillagigedo, Michoacan, Costa Rica, and Galapagos-Machalilla [6, 10]. Most of these MUs have been proposed based on genetic data exclusively from nesting sites [6]. Just for the case of Galapagos and Northwest Hawaii genetic data from foraging grounds have also been considered [10, 57].

Our results based on foraging grounds showed well differentiated groups for Costa Rica, Galapagos and New Zealand. Thus, although the natal origin (EPGL and WPGL) had great relevance in the morphological differentiation of populations, as previously discussed, foraging sites also could have an effect on carapace shape variation in this species.

Previous studies in chelonians have shown an association between environmental conditions and the carapace shape, which has been attributed to both, natural selection and phenotypic plasticity [31–33, 58–61]. For instance, shell shape variation has been related to different flow regimes (lentic vs lotic) [31, 62], habitat types [60, 63], lifestyles (e.g. digging ability) [59], predation pressure [61, 64], movement patterns (migrant and non-migrant) [63] and thermoregulation [58] in tortoises and freshwater turtles.

Although empirical fitness data would be required to properly asses the adaptive value of the carapace shape in *C*. *mydas*’ lineages, further research examining the relationship between carapace morphology, specific environmental conditions in foraging grounds, and non-neutral genetic variation, may provide more insight about selection pressures on the carapace shape of this species. In this context, and given the longevity and conservation status of *C*. *mydas* that restrict experimental studies, genomic tools may be useful to address these questions.

Regarding Chile, the lack of population differentiation could be due to Chilean waters just constituting foraging habitats for *C*. *mydas* (no nesting exists) and the natal origin of individuals is mainly Galapagos-Ecuador [37]. The latter, is supported by the low differentiation between both populations (Chile-Galapagos, Fig 4 and Table 3). In any case, research increasing the sample size in Chile could allow us to observe a clearer pattern of morphological variation.

Our results based on foraging grounds reveal the importance of studying the role of selection on the morphology of *C*. *mydas*, and the relevance to incorporate environmental and genetic information from these habitats when a MU is defined. In this way, by integrating data from the key habitats of the green turtle’s life cycle, the evolutionary potential of their threatened populations can be preserved.

## Conclusions

Our study shows that the carapace shape in *C*. *mydas* is markedly associated with the species’ lineages suggesting a substantial genetic influence on this trait. Based on the relationship between carapace shape and genetic lineages found here, we propose the existence of at least three distinct morphotypes of *C*. *mydas*: Atlantic, Eastern Pacific and Western Pacific. Well-differentiated groups in some foraging grounds may suggest an effect of ecological or environmental operating conditions on morphological variations of *C*. *mydas*. Likewise, these results highlight the importance to integrate data from rookeries and foraging grounds to define MUs in order to conserve the evolutionary potential of distinctive populations. This is the first study using geometric morphometrics to evaluate the congruence between phylogeography and morphological variation in marine turtles. Our results, based on this emergent tool, open new possibilities to test ecological and evolutionary hypotheses in morphologically variable and widely distributed species.

## Supporting information

S1 Table List of individuals used in this study including foraging ground (country), specific location, year of collection and genetic lineage or natal origin (haplotype origin). (XLSX)

S1 Fig Difference in carapace shape between *Chelonia mydas* from different genetic lineages. **Scatterplot of first two axes of the principal component analysis.** Eastern Pacific (EPGL): black; Western Pacific (WPGN): yellow, and Atlantic (AGL): red. * All the analyses have size effect removed.
(JPG)
