# Supplementary figures and images for: Identifying genetic lineages through shape: An example in a cosmopolitan marine turtle species using geometric morphometrics
Source: PLoS One. 2019 Oct 7;14(10):e0223587. doi: 10.1371/journal.pone.0223587 (PMC6779254; doi:10.1371/journal.pone.0223587)

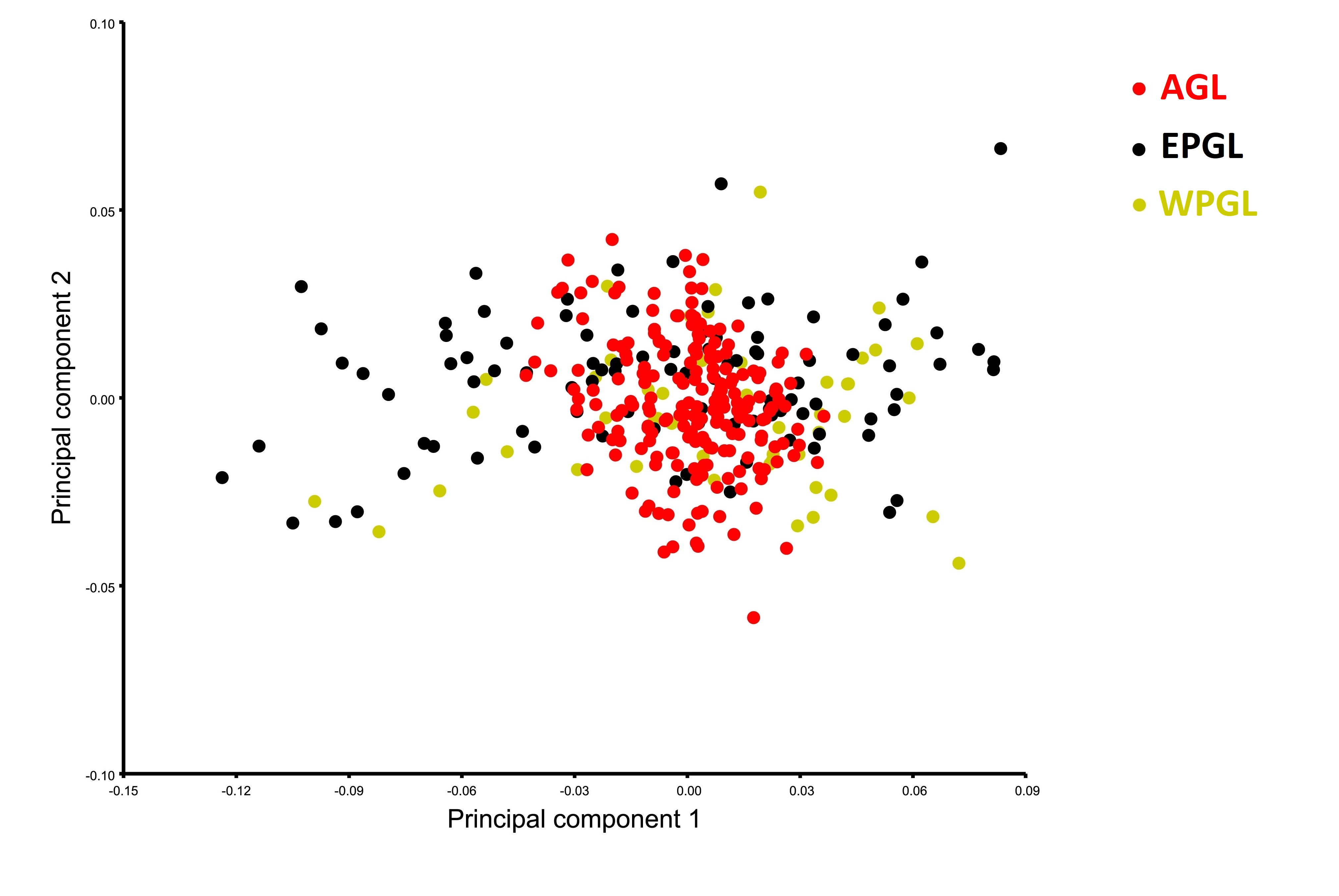

Supplement: S1 Fig — Scatterplot of first two axes of the principal component analysis. Eastern Pacific (EPGL): black; Western Pacific (WPGN): yellow, and Atlantic (AGL): red. * All the analyses have size effect removed. (JPG) [file pone.0223587.s002.jpg]
